# An art-based labyrinth activity workshop experience: a qualitative study on psychological counselor candidates from the perspective of acceptance and commitment therapy

**DOI:** 10.3389/fpsyg.2026.1872185

**Published:** 2026-07-06

**Authors:** Gamze Mukba

**Affiliations:** Van Yüzüncü Yıl University, Van, Türkiye

**Keywords:** acceptance and commitment therapy processes, art-based labyrinth activity workshop, deductive content analysis, psychological counselor candidates, qualitative descriptive research, well-being

## Abstract

**Purpose:**

Art-based activities provide a flexible, nonverbal medium for exploring emotions, personal values, and inner experiences. Within the framework of acceptance and commitment therapy (ACT), art-making has been discussed in relation to psychological flexibility processes, such as acceptance, cognitive defusion, self-as-context, and value-based action. This study aimed to explore psychological counselor candidates’ descriptions of their cognitive, emotional, and behavioral experiences related to participation in an art-based labyrinth activity workshop from an ACT perspective.

**Methods:**

This study employed a qualitative descriptive research design with 81 psychological counselor candidates (53 females and 28 males) who were final-year undergraduate students in the Department of Psychological Counseling and Guidance at Van Yüzüncü Yıl University. Participants engaged in an art-based workshop utilizing a symbolic labyrinth technique and completed written reflections on their experiences. A deductive content analysis approach was employed for data analysis, as the codes, subcategories, and categories derived from the data were interpreted within the framework of Acceptance and Commitment Therapy (ACT).

**Results:**

The findings indicated that the participants’ reflections corresponded with the ACT processes. Under the category of “contact with the present moment and acceptance,” the subthemes “emotional relief” and “emotional confrontation” emerged. Within “self-as-context,” the subcategories included “self-esteem and inner observer,” “reconciliation with personal experiences and the self,” and “self-awareness.” The “values” category comprised “valuing challenges and solutions,” “assigning value to emotions,” “valuing experiences and actions related to family members,” and “attributing value to early childhood memories––first toy.” Under “defusion,” the subcategories “cognitive defusion––a new perspective on thoughts” and “emotional defusion––a new perspective on emotions” were identified. Finally, the “committed action” category encompassed the subcategories “decisive steps” and “a new journey.” Overall, the participants’ reflections suggested categories related to openness toward internal experiences, metaphorical meaning-making, and value-based reflections.

**Conclusion:**

The candidates’ narratives suggested that the art-based labyrinth activity workshop provided a reflective and experiential context for exploring emotional shifts, values related to important personal contexts and situations, and meaning-making processes. The findings highlight the potential relevance of art-based activities for experiential reflection, self-exploration, and well-being-related reflection in counselor education and supervision contexts.

## 1 Introduction

This study explored the emotional, cognitive, and experiential reflections of counselor candidates on art-based labyrinth activity workshops through the lens of Acceptance and Commitment Therapy (ACT) processes, including self-reflection, emotional burdens, values, and meaning-making experiences (Kimball, 2018; Sanders, 2016; Zubala et al., 2014). Art-based activities may provide opportunities for symbolic expression and emotional reflection through visual forms such as drawing and creative representation (Çelikbaş, 2019; Dilawari and Tripathi, 2014). Such practices may also be considered a creative dialectical intersubjective paradigm that addresses individuals’ ways of being, interpersonal processes, and meaning-making experiences that emerge through educational and experiential contexts (Gerber, 2026). Rogers (1993) conceptualized person-centered, expressive, arts-based activities as creative and non-directive practices grounded in empathy, authenticity, and trust in the individual’s inner capacity for growth. In postmodern life, creative practices may provide opportunities for narrative exploration and interpretation of interpersonal experiences through metaphorical and experiential forms of communication (Darewych, 2022; Karkou et al., 2011; Nelson et al., 2024; Tompkins Rosa, 2023).

Creative arts-based activities can be implemented in various forms, including expression through painting, music, and movement or dance (Carr et al., 2023; Chilton et al., 2021; Kelly et al., 2015; O’Callaghan and Aasgaard, 2012). Creative arts-based approaches, such as music, dance/movement, and visual art practices, are related yet distinct fields that involve different expressive modalities and professional frameworks (Edwards et al., 2020; Karkou et al., 2011). Creative arts-based approaches such as music and dance/movement-based practices are often more frequently represented in rehabilitation and health-related contexts, whereas visual art-based practices tend to emphasize symbolic expression, reflection, and narrative exploration (Edwards et al., 2020; Lee et al., 2025). Accordingly, this study focused on an art-based labyrinth activity emphasizing visual reflection and the description of participants’ emotional and interpersonal experiences through narrative expression (Edwards et al., 2020; Weinfeld-Yehoudayan et al., 2024).

Art-based activities have been discussed in relation to emotional experiences, reflective processes, and coping-related expressions among individuals participating in expressive practices (Haeyen and Noorthoorn, 2021; Han, 2023; Kowitt et al., 2016; Nan et al., 2021). Moreover, such practices have been explored in relation to cognitive, emotional, and behavioral experiences in socio-culturally rooted contexts, including migration, war, and peer bullying among children (Feen-Calligan et al., 2023; Lobban and Murphy, 2019; Yan et al., 2019). Professionals engaging in art-based activities, such as psychological counselors, may also participate in experiential processes involving reflection on emotions, interpersonal perspectives, and nonverbal forms of emotional expression (Boiadjieva, 2021; Deaver and Shiflett, 2011; Partridge, 2021). For psychological counselor candidates, firsthand engagement in such activities may involve reflections on emotional experiences, interpersonal processes, and professional perspectives (Batık and Talay, 2020; Gökkaya, 2022; Yıldız and Toprak, 2016).

Consistent with the self-practice or self-reflection framework proposed by Bennett-Levy and Lee (2014), creative and experiential techniques may also facilitate reflection on personal meanings, self-related perceptions, and distinctions between the self and others for psychological counselors (Barton, 2020; Carden et al., 2023). Furthermore, reflective self-integration may serve as a lens through which psychological counselors describe and interpret their own internal experiences and those of their clients (Nissen-Lie et al., 2020; Pérez-Rojas et al., 2017).

Psychological counselor candidates may benefit from becoming familiar with different expressive and reflective approaches that can be adapted to individuals’ unique characteristics and communication styles (Norcross and Wampold, 2019). Exposure to multiple perspectives may also contribute to their awareness of how art-based activities can be incorporated into educational, developmental, and reflective contexts with children and adults (Cui and Wang, 2022; Potash et al., 2012). For example, counselors may invite children to reflect on their experiences through prompts such as, “How did participating in this activity make you feel?” or “What was it like to share your drawing with the group?” (Yuen, 2004, pp. 464–465). Through artistic expression, individuals may describe emotions, attribute meaning to experiences, reflect on interpersonal interactions, and engage with emotional experiences through symbolic and nonverbal forms of communication (Chapman and Evans, 2020; Kavaklı, 2019).

Accordingly, art-based activities and practices may provide opportunities for meaning-making processes through which individuals reflect on new actions and different perspectives for understanding, interpreting, and responding to experiences from the view of ACT (Backos, 2023; Hayes and Rowse, 2008). In this study, an art-based labyrinth activity workshop was designed as an experiential inner journey focused on reflections related to experiences conceptually associated with ACT processes. The symbolic use of selecting, carrying, releasing, and re-collecting stones representing emotional burdens and newly gained meanings was associated with experiences interpreted through the lens of ACT processes: (1) contact with the present moment, (2) acceptance, (3) cognitive defusion, (4) values, (5) values-based reflection, and (6) committed action (Zhang et al., 2018). The inward journey symbolically represented reflections related to emotional difficulties and experiences of letting go, whereas the return journey was associated with meaning-making experiences related to self-as-context, including trust, insight, strength, and self-growth (Tayyebi et al., 2024). During the inner journey, emotional burdens and difficulties may be reflected upon as experiences that do not fully define the self but can instead be interpreted as parts of one’s ongoing experiences and meaning-making processes (Balta et al., 2025). In this context, emotion-related metaphors and experiential exercises, such as reflecting on metaphors and describing them from different perspectives while remaining in contact with the present moment, have been discussed in relation to ACT processes associated with emotional distancing, reflection, and value-based meaning-making (Blackledge and Hayes, 2001; Mollica, 2022; Mueller and Silvestre-López, 2026).

Within the frameworks of ACT and positive psychology, personal descriptions and meaning-making experiences related to psychological flexibility and values may also provide a perspective for understanding well-being-related experiences (Kroemeke et al., 2022). Accordingly, the central research question in this study is: “How do psychological counselor candidates describe their cognitive, emotional, and behavioral reflections following an art-based labyrinth activity workshop interpreted through an ACT lens?” This study also aimed to contribute to experiential approaches in counselor education and to positive psychology perspectives emphasizing meaning-making, personal values, and reflective exploration of well-being-related experiences.

### 1.1 Art-based practices and psychological flexibility

Art-based activities are creative and expressive practices that may support individuals in exploring their self-perceptions, emotions, and thoughts through symbolic forms of expression. These activities may facilitate reflective and descriptive processes in which individuals observe themselves from different perspectives and engage with emotional experiences without relying solely on verbal language (Blomdahl et al., 2013; De Witte et al., 2021; Franklin, 2012; Rankanen, 2014). For instance, in grief and loss contexts, acceptance-oriented and art-based activities have been described as providing opportunities for emotional expression and reflection related to mourning experiences (Davis and Tungol, 2019).

Through art-based activities, individuals can nonverbally and flexibly engage with personal values and life possibilities, including both desired outcomes and situations they wish to avoid, without explicit prompting (LeBaron, 2011). These possibilities are shaped by contextual interactions involving the self, others, and the environment (Fantinelli et al., 2023; Lent et al., 2000; Rigoli et al., 2016). These activities may also be conceptually interpreted through an ACT lens, including processes related to (1) contact with the present moment, (2) values, (3) acceptance, (4) cognitive defusion, (5) self-as-context, and (6) committed action (Hayes et al., 2006; Hayes et al., 2012; Kul and Türk, 2020).

“Being present” refers to mindful engagement with the present moment, free from fixation on past or future events (Kul and Türk, 2020, p. 3785; Zhang et al., 2018, p. 4). “Values” are internal motivators that guide meaningful action and help individuals determine who they want to be (Hayes et al., 2006, p. 9; Kul and Türk, 2020, p. 3787). “Acceptance” involves allowing distressing emotions, memories, and urges to exist without resistance. “Cognitive defusion” refers to observing thoughts and feelings as inner experiences rather than literal truths (Hayes et al., 2012, pp. 982–983; Zhang et al., 2018, p. 4). “Self-as-context” allows individuals to recognize a stable sense of self that is independent of transient experiences. “Committed action” represents the pursuit of value-based behaviors with flexibility and persistence (Hayes et al., 2006, pp. 9–10; Hayes et al., 2012, p. 984).

Art-based activities may provide reflective contexts in which experiences related to these processes may emerge naturally. Artistic expression may provide opportunities for individuals to describe and reflect on their internal experiences, personal values, and present-moment awareness through symbolic and nonverbal forms of expression (Dudley et al., 2014; Schweizer et al., 2017). Such experiences may also involve reflections related to self-other distinctions, emotional awareness, and interpersonal understanding (Burt, 2002; Sherman and Morrissey, 2017; Feen-Calligan et al., 2020; Slayton et al., 2010).

Furthermore, art-based activities have been associated with value clarification (Reilly et al., 2021; Talwar et al., 2004), reflections related to “self-worth” (Simoneaux, 2011, p. 159), and increased awareness of emotional and behavioral patterns (Blomdahl et al., 2013; De Witte et al., 2021; Franklin, 2012). These experiences are conceptually consistent with psychological flexibility, which involves openness to internal experiences and engagement in actions aligned with personally meaningful values (Assaz et al., 2023; Hayes et al., 2006). In this context, participants’ written reflections in art-based activities may also be interpreted through an ACT lens (Backos, 2026).

## 2 Method

### 2.1 Research design

This study employed a qualitative descriptive research design to explore reflections and experiences of psychological counselor candidates regarding an art-based labyrinth activity workshop. Qualitative descriptive research aims to provide a detailed and experience-oriented understanding of participants’ subjective reflections and interpretations in a particular context (Kim et al., 2017). Consistent with this descriptive and experience-focused approach, this study explored participants’ written reflections within the ACT conceptual framework.

The data were analyzed using deductive qualitative content analysis guided by the six core ACT processes. Qualitative content analysis can be conducted using inductive and/or deductive reasoning, depending on the research aim and theoretical framework (Bengtsson, 2016). Directed qualitative content analysis is particularly appropriate when existing theories or conceptual frameworks are used to guide the coding and interpretation processes (Assarroudi et al., 2018). In this respect, deductive qualitative inquiry enabled the researcher to descriptively organize and interpret participants’ subjective experiences through pre-existing conceptual structures related to ACT processes (Robinson and Bailey-Rodriguez, 2026; Dahal, 2025).

In this study, participants’ written reflections were descriptively organized with reference to an ACT-oriented conceptual framework that included acceptance, defusion, contact with the present moment, self-as-context, values, and committed action. Accordingly, these ACT processes were used as sensitizing conceptual categories during the analysis, rather than as rigid or exclusive coding structures. This approach provided a flexible framework for descriptively exploring how participants reflected on their emotional experiences, metaphorical meaning-making processes, values, goals, and personal experiences related to the art-based labyrinth activity workshop.

### 2.2 Participants

The participants of this study consisted of 81 final-year undergraduate students from the Psychological Counseling and Guidance Department at Van Yüzüncü Yıl University, including 53 females and 28 males. The participants were enrolled in a four-year undergraduate training program in the Psychological Counseling and Guidance Department. All participants volunteered to participate in the study. The mean age of the participants was 24.13 years. The researcher announced the art-based workshop activity to final-year psychological counselor candidates at Van Yüzüncü Yıl University and informed the students that participation was entirely voluntary. In accordance with the purposive sampling approach, the research specifically focused on students enrolled in the final year of the undergraduate program in Psychological Counseling and Guidance, because they had completed most of their professional counseling training and were considered capable of engaging in reflective and experiential activities related to psychological counseling processes. The researcher was involved in teaching courses attended by final-year undergraduate students in the Department of Psychological Counseling and Guidance who participated in the study. However, the workshop was not conducted within the scope of any formal course requirements or academic evaluation processes. Participants were informed about the experiential and reflective nature of the workshop prior to participation. The relatively high level of participation may reflect psychological counselor candidates’ educational and professional interest in experiential and reflective workshop activities. Written and verbal informed consent was obtained from all participants prior to data collection.

The participants had not received formal training in ACT, expressive arts methods, or art-based therapeutic practices before the workshop. Moreover, none of the participants had previously participated in a structured ACT- or art-based experiential workshop. However, as final-year undergraduate students in psychological counseling and guidance, they had prior exposure to basic counseling theories, counseling skills, and undergraduate counseling practice courses in their professional education.

The sample size of 81 participants was considered appropriate for the qualitative descriptive design of the study, which aimed to explore a broad range of reflections and experiential responses related to the art-based labyrinth activity workshop. Using written reflective narratives together with deductive qualitative content analysis was considered appropriate for enabling an in-depth interpretation of participants’ experiential reflections (Fife and Gossner, 2024) within an ACT-oriented conceptual framework. Data depth was supported through detailed written reflections focusing on emotional experiences, metaphorical meaning-making processes, and psychological flexibility-related experiences that emerged during the workshops.

### 2.3 Procedure

The implementation process of this study was carried out in the following steps.

#### 2.3.1 Step 1: formulating the research question

From a reflexive and descriptive perspective, the researcher approached the study to understand how experiential and art-based reflective activities relate to emotional awareness, self-reflection, and meaning-making processes associated with the core processes of ACT among psychological counselor candidates. The facilitator/researcher also participated in training programs focused on storytelling and fairy tale-based approaches applicable to children and adults within expressive and creative practice contexts and had previous practical and professional experience in these areas. The researcher also participated in a training program that included the self-image model (Lebedeva, 2014) and the labyrinth technique, which uses symbolic movement and metaphors to support emotional expression. Inspired by this approach, the researcher aimed to explore how psychological counselor candidates might experience and reflect on their inner emotional and cognitive processes when engaged in an art-based labyrinth activity. According to Lebedeva (2012, 2014), art-based activities may facilitate the expression of hidden anger, trauma, and emotional conflict through forms of self-representation and metaphor, allowing opportunities for emotional exploration, reflection, and new meaning-making experiences.

#### 2.3.2 Step 2: developing the workshop protocol

The facilitator/researcher had completed approximately 20 h of training (Lebedeva, 2017) related to self-image art-based techniques, such as art-based labyrinth activity and experiential reflective practices. Moreover, the facilitator had prior experience using art-based and metaphor-oriented experiential practices within counseling-related processes and workshop settings involving psychological counseling candidates.

A debriefing-oriented informational framework regarding the experiential and emotional reflective nature of the activity was provided before the workshop. The participants were informed that emotionally evocative themes and personal reflections might emerge during the workshop process. They were also reminded that when they felt emotionally overwhelmed or uncomfortable, they could use breathing exercises and “here-and-now” grounding awareness to regulate and calm themselves. Moreover, participants were informed that if feelings of emotional discomfort persisted after the workshop, they could seek support from the university’s psychological counseling center. The facilitator remained available throughout the workshop process to provide emotional support in the event of any acute distress or crisis-related emotional reactions and stayed with the participants during the activities.

The art-based labyrinth activity workshop was designed based on Prof. Dr. Lebedeva’s (2017) original instructions for the labyrinth technique, which encourages the symbolic exploration of inner emotional states. Participants were first guided to recall a past conflict they wished to release and select symbolic stones representing associated emotions, such as anger, fear, sadness, or resentment. Using modeling clay, each participant created a puppet to carry these symbolic burdens through a printed labyrinth figure. To make the labyrinth suitable for a modeling clay puppet to move through, the researcher reviewed online sources and prepared a finalized version of the labyrinth for each participant on an A4 sheet. The researcher adapted the instructions (Lebedeva, 2017) to include the following reflective guidance:

*“Choose stones that represent the emotions you wish to release. How do you feel about this? What emotions do these stones carry for you? What do you want to replace them with? During your journey through the labyrinth, you will leave these stones behind. On the way back, you will collect different stones representing what you have learned and gained, such as trust or experience. Give this journey a name and reflect on what you felt.”*

Participants were encouraged to interpret the metaphor freely and explore their own symbolic journeys of letting go and reflections related to meaning making. The researcher prepared all session materials in advance, including printed labyrinth figures and modeling clay, ensuring that each component was suitable for the guided activity.

#### 2.3.3 Step 3: ethical approval

Prior to data collection, ethical approval was obtained from the Ethics Committee of Van Yüzüncü Yıl University and the Social and Human Sciences Publication Ethics Committee (Decision No: 2024/10-60, Date: May 23, 2024). The ethical approval process covered both the experiential workshop activity and the collection of participants’ written reflections related to their experiences. Prior to the workshop, participants were informed that the art-based labyrinth activity could involve reflection on emotionally difficult experiences and personal memories. The participants were informed about the purpose of the study before the workshop activity, and written informed consent was obtained for participation and for the publication of artwork images included in the study. All artwork images were reviewed to ensure that they did not contain any identifying personal information.

#### 2.3.4 Step 4: art-based labyrinth activity workshop

The workshops were conducted with undergraduate students in the Psychological Counseling and Guidance Department of Van Yüzüncü Yıl University. The workshop was conducted in a classroom setting. The participants were informed about the importance of respecting personal boundaries, confidentiality, and privacy within the group process before the activity. Participants were not required to share personal experiences or emotional reflections during the workshop. Participants were asked to focus on their individual inner journeys without speaking or verbally sharing personal material with other group members. Written reflective narratives were completed individually, and participants were instructed not to engage in personal sharing with one another during the workshop process. Moreover, participants were informed that they had the right to refrain from discussing personally or emotionally sensitive experiences throughout the study.

Two separate implementations were carried out: one with psychological counselor candidates studying in the Spring term of the 2023–2024 academic year and the other with those studying in the Fall term of the 2024–2025 academic year. The workshop sessions were conducted in June and December 2024 in a designated classroom setting. The researcher informed the participants about the purpose of the study and obtained written informed consent. Each participant received modeling clay and a printed labyrinth figure. After receiving the instructions, the participants engaged in the activity for approximately 70–80 min.

#### 2.3.5 Step 5: data collection

The primary data source of the study consisted of participants’ individually completed written reflective narratives, which formed the basis for the deductive qualitative content analysis. Visual materials were collected only as metaphorical representations related to the experiential activity process. In art-based activities, visual materials may serve as supportive and complementary elements that facilitate deeper reflective meaning-making within written narratives, rather than constituting the primary source of analysis (Fabris et al., 2023; Ho et al., 2021). Moreover, visual materials in art-based activities may provide individuals with alternative ways of expressing emotional experiences beyond words and written language and support emotional expression and relief processes (Bergbom and Lepp, 2022).

A limitation of the data collection process is that the present study relied exclusively on written reflections collected immediately after the workshops. Nevertheless, reflective writing is considered a valuable qualitative data source for exploring subjective experiences, personal meanings, and reflective learning processes (Harper, 2013; Olmos-Ochoa et al., 2021). Written reflections allow participants to express their thoughts in their own words and at their own pace, which may facilitate deeper self-reflection and meaning-making following experiential and psychologically oriented workshop activities (Olmos-Ochoa et al., 2021). However, as no follow-up interviews or further opportunities for clarification were conducted, some experiences may not have been explored in the same level of detail that could be achieved through more interactive qualitative methods (Harper, 2013).

It should be acknowledged that the reflective prompts encouraged participants to consider their emotional experiences, perceived changes, and the personal meanings associated with the workshop. Therefore, the structure of the prompts may have influenced the aspects of the experience emphasized in the reflections. However, consistent with the interpretive nature of qualitative research, the findings emerged through participants’ subjective interpretations and meaning-making processes, allowing individual realities and personal perspectives to shape the content of their reflections (Harper, 2013; Ooi et al., 2023). The participants completed all written reflections in Turkish. The reflections were analyzed in their original language, and the quotations presented in this manuscript were translated into English by the researcher during the analysis and manuscript preparation processes. Particular attention was paid to preserving the intended meaning, contextual nuances, and original style of expression in written reflections during translation.

The researcher developed the written interview questions used in this study. Immediately after the workshop, participants were asked to respond in writing to a set of open-ended questions. These included: *“What did you experience during this activity?” “Which emotional burdens did you release, and what new feelings did you gain?” “Please describe this journey as it relates to your inner world and give your journey a title. Tell this journey as a story in which you are the main character. The title you provide can also reflect your emotions and the points of change or transformation you noticed during the journey.”* Participants engaged in the art-based labyrinth activity workshop for approximately 70–80 min. Following the experiential activity, participants were given 50–60 min to complete their written reflections by hand on A4 paper, responding individually in a descriptive and narrative style. Accordingly, the entire workshop and data collection process lasted approximately 120–140 min in total. The instructions for the Written Reflection Form are presented in the Supplementary material (see Supplementary File).

The researcher reviewed the relevant literature and structured the written instructions by drawing inspiration from the art-based activities training received from Lebedeva (2017) as well as from previous research (Öster et al., 2007; Slayton et al., 2010). For instance, Öster et al. (2007) structured interview questions to examine the emotions, thoughts, and experiences of participants diagnosed with cancer during art-based activities, focusing on coping resources, quality of life, symptoms, and self-image. Additionally, participants were encouraged to express their experiences and emotions through weekly notes and journals kept throughout the art-based sessions (Öster et al., 2007). In art-based activities, the ways participants make sense of their experiences may provide opportunities for describing and reflecting upon emotional and cognitive experiences through physical and expressive processes. In this context, as highlighted by Slayton et al. (2010), participants’ feelings, thoughts, and descriptions of their experiences may be explored in greater depth through flexibly structured questions used during art-based activities and reflective practices.

### 2.4 Data analysis

In this study, a deductive content analysis approach was used, as the codes, subcategories, and categories derived from the data were interpreted in relation to a pre-established theoretical framework, referred to as ACT, a model of psychological flexibility (Hsieh and Shannon, 2005; Patton, 2014). The written reflections produced by psychological counselor candidates during the art-based labyrinth activity sessions were originally handwritten on A4 paper. The handwritten reflections were manually transferred into a digital format by the researcher and reviewed, organized, and coded during the deductive qualitative content analysis process. The texts were read multiple times to gain a holistic understanding of the participants’ cognitive, emotional, and behavioral experiences.

Initial codes were identified based on meaningful units in participants’ statements. An initial coding booklet was developed by the researcher based on the six core ACT processes: contact with the present moment, connection with values, committed action, cognitive defusion, self-as-context, and acceptance (Aslan and Kabasakal, 2024). The booklet included ACT dimensions, preliminary subcategories, and representative codes intended to guide the deductive coding process. Throughout the analysis, the coding booklet was reviewed and refined iteratively in response to emerging patterns in the participants’ reflective narratives. Coding was conducted at the meaning-unit level. Participants’ written reflections were segmented into meaningful units and assigned to ACT categories according to their dominant psychological function and conceptual fit with the coding manual. For instance, statements reflecting emotional awareness, emotional acknowledgment, and willingness to engage with difficult emotions were categorized under contact with the present moment and acceptance. Statements reflecting self-observation, self-awareness, self-esteem, or reconciliation with personal experiences were categorized as self-as-context. Expressions emphasizing personally meaningful experiences, emotions, relationships, or life directions were categorized under values. Statements demonstrating a shift in perspective toward thoughts or emotions were categorized as defusion, whereas statements reflecting decision-making, behavioral commitment, or movement toward valued directions were categorized as committed action.

Although conceptual overlap between ACT processes was acknowledged, meaning units were assigned to the category that best reflected their dominant function within the participant’s narrative and the overall contextual meaning. The frequencies reported for subcategories represent the number of participants whose narratives contained at least one meaning unit corresponding to the relevant subcategory. Since participants could describe experiences related to multiple ACT processes, a single participant could be represented by more than one category or subcategory.

Following the completion of the coding process, the coding booklet, including the resulting codes, subcategories, and categories, was reviewed in consultation with a domain expert specializing in psychological counseling and ACT (Chen et al., 2026; Haring et al., 2020). The expert provided feedback on the conceptual alignment of the coding structure with the ACT framework, particularly concerning the categorization of codes and subcategories within the psychological flexibility processes. This feedback was incorporated into the final coding structure to enhance conceptual consistency and theoretical coherence. For instance, the subcategories “Emotional Relief” and “Emotional Confrontation” were found to reflect both present-moment awareness and acceptance processes, as the participants’ reflections frequently involved simultaneously contacting and acknowledging emotional experiences. Based on discussions between the researcher and the expert, these overlapping elements were integrated into the final coding structure, thereby strengthening the theoretical coherence and interpretive consistency of the analysis. Similar codes were merged and redefined to form coherent subcategories, which were then organized under theoretically informed categories aligned with the core ACT processes.

This study employed an art-based activity rather than a structured art therapy intervention. The activity was used as a reflective and experiential tool to facilitate engagement with ACT processes rather than as a therapeutic modality. As mentioned previously, the researcher had previously completed approximately 20 h of workshop training in art-based activities, including the art-based labyrinth activity used in the present study. This training informed the implementation of the activity and supported the interpretation of the participants’ reflective narratives. As the art-based labyrinth activity was used as a reflective and experiential exercise rather than a therapeutic technique, no further consultation with an art therapy specialist was sought.

Reflexivity is conceptualized as an ongoing process of critically examining the researcher’s role, assumptions, relationships, and interpretive decisions throughout the research process (Evans et al., 2018; Muthanna and Alduais, 2023). In line with qualitative approaches that emphasize researcher positionality, transparency, and continuous reflection, reflexive practice was employed to maintain focus on the purpose of the study, participants’ perspectives and experiences, and the meaningful interpretation of their reflective narratives (Evans et al., 2018; Muthanna and Alduais, 2023). The researcher served as both the facilitator of the art-based activity and the primary analyst of the qualitative data. The participants were undergraduate students within the same academic department and were therefore familiar to the researcher through teaching activities and academic interactions. As described previously, the activity was conducted outside the context of course requirements and participation was entirely voluntary. The researcher approached the study with the understanding that art-based reflective activities may facilitate engagement with ACT processes; however, interpretations were grounded in participants’ written reflections and narrative accounts throughout the analytic process.

Throughout the study, reflexivity served to maintain focus on the participants’ accounts, support transparency in analytic decision-making, and encourage continuous critical examination of the researcher’s interpretations and relationship to the data (Evans et al., 2018). Accordingly, the researcher repeatedly revisited the coding booklet, codes, subcategories, and categories to evaluate their conceptual fit, consistency, and alignment with the participants’ written reflections and narrative accounts (Braun and Clarke, 2024). Consequently, analytic decisions and subcategory assignments were continually reviewed together with the ACT expert to maintain alignment with the study aims, conceptual coherence within the ACT framework, and fidelity to participants’ narratives.

### 2.5 Trustworthiness and credibility of the study

Lincoln and Guba (1985) identified several criteria for evaluating the trustworthiness of qualitative research: credibility, dependability, confirmability, transferability, and authenticity. Credibility refers to the accurate representation of participants’ perspectives and a clear description of the research process. Dependability involves the stability and consistency of the findings over time and under different conditions. Confirmability addresses the objectivity and relevance of the data, which can be supported through the inclusion of direct quotations. Transferability relates to the applicability of the findings to other settings or groups, whereas authenticity concerns the extent to which the research fairly represents participants’ experiences. In this study, the research context, participant group, analytic procedures, coding processes, and iterative development of the coding booklet are described in detail to support transparency, reflexivity, and methodological coherence throughout the qualitative inquiry (Braun and Clarke, 2023; Cypress, 2017). To strengthen the reliability of the data analysis, expert consultation was obtained from a scholar in psychological counseling and ACT (Lincoln and Guba, 1985; Pandey and Patnaik, 2014).

After the workshop, participants were invited to write a reflective narrative on an A4 sheet in response to the prompt, “*Describe your experience during this art-based activity session. What emotional burdens did you experience? What new emotions did you experience? Please give your journey a title*.” These narratives represent the participants’ personal interpretations of the workshop experience and are consistent with the goal of qualitative inquiry, which seeks to explore the meaning that individuals assign to a given experience (Kvale, 1994; Polkinghorne, 2005).

Participants provided written accounts and integrated symbolic visual elements (such as their labyrinth drawings), providing data in both verbal and nonverbal formats. Their engagement in the “here and now” through the creative art-based process enhanced the authenticity and originality of the data collection (Csikszentmihalyi, 1990; Polkinghorne, 2005). The combination of written reflections and visual expressions allowed for a rich, self-reflective dataset (Bateman and Wildfeuer, 2014; Pinnegar and Hamilton, 2009), contributing to both the credibility and authenticity of the study. Supporting the findings with direct quotes and aligning the emerging themes with the ACT framework through deductive content analysis further enhanced the confirmability and transferability of the results (Bell, 2001; Erfani, 2021; Elo et al., 2014).

## 3 Findings and results

**Figure 1:**
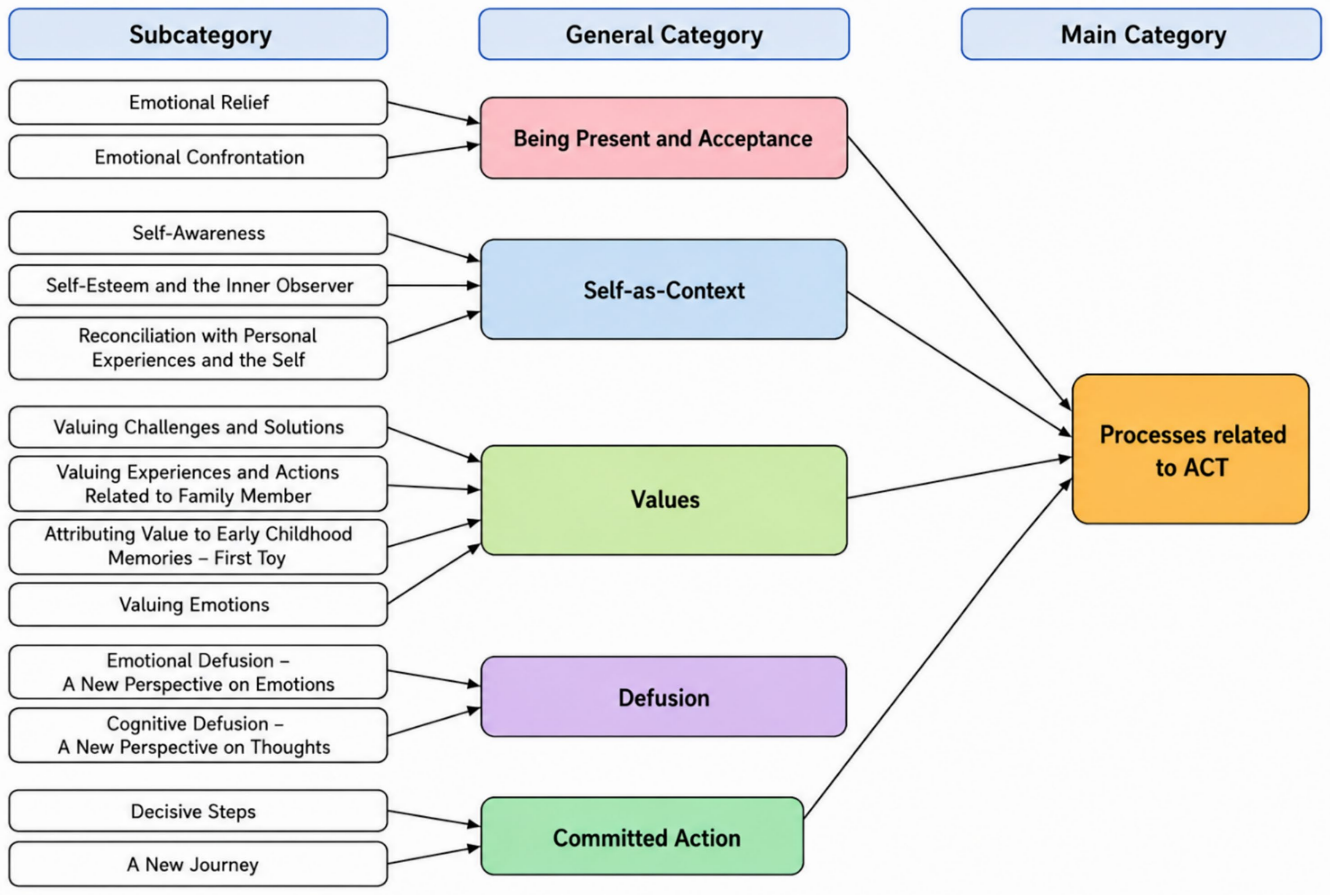
Main category, general categories, and subcategories based on the processes related to ACT.

Main category, general categories, and subcategories identified based on the processes related to ACT are presented in Figure 1. The categories and subcategories, along with representative quotations from participants, are presented in Table 1.

**Table 1:** General categories, subcategories, and illustrative quotes emerging from the participants’ art-based workshop experiences, organized according to processes related to ACT.

General category	Subcategory	*n*	Illustrative quote
Being present and acceptance	Emotional relief	25	P1 (Woman): “Letting things go during this journey was quite difficult. But it was necessary for healing (.).”
	Emotional confrontation	63	P80 (Woman): “My emotions told me: Anger: ‘I will be with you throughout your life.’ Fear: ‘I stay with you even more because you are afraid.’ Disappointment: ‘I am always in your heart, I will never leave.’.”
Self-as-context	Self-awareness	10	P81 (Woman): “After going to the center and coming back, I became aware that I needed to leave behind the burdens on my back. Yes, those burdens are ‘my self’, but they are also something that made me question my being- something that reminded me that I had to stop somewhere, and that I had to continue my life no matter what…”
	Self-esteem and the inner observer	25	P10 (Man): “The puppet’s success story… Belief in itself… Our puppet is now happier, believes in itself, and has full confidence that it can overcome challenges.”
	Reconciliation with personal experiences and the self	5	P74 (Man): “I realized that I needed to live with disappointment more consciously and peacefully (.) I understood that I should no longer expect everything from everyone.”
Values	Valuing challenges and solutions	27	P74 (Man): “My anger and disappointment will exist in my life only if I allow them to. I’ve learned not to reflect (disappointment) onto everyone like I used to, and to use it in a more measured way. I used to tell my loved ones what they did wrong and that would bring me peace.”
	Valuing emotions	5	P22 (Man): “, I think compassion allows me to see the pain or distress someone else is going through. I also think I feel good when I help, and I believe it makes the other person feel better too.”
	Valuing experiences and actions related to family members	3	P80 (Woman): “Maybe my father was very tired that day, and that’s why he got angry. So, my feeling of anger turned into empathy. I thought to myself that maybe the way he was raised caused him to react that way.”
	Attributing value to early childhood memories––first toy	1	P49 (Woman): “I’ve always told you about my firsts, because I believe firsts are always important in a person’s life.”
Defusion	Cognitive defusion––a new perspective on thoughts	13	P32 (Woman): “Indecisiveness: ‘Trying different paths turned into a solution for me.’ As for helplessness, she changed it with the thought that there is always a way”
	Emotional defusion––a new perspective on emotions	33	P26 (Woman): “The fear stone (…), I transformed into excitement. Excitement: ‘You should not be afraid of new ventures. You should let yourself go freely into the future.’”
Committed action	Decisive steps	11	P63 (Woman): “‘Now it’s time to strive for your own desires.’ Throughout my life, I’ve worked hard for my family and what they wanted. Actually, since our wishes mostly aligned, it wasn’t a struggle.”
	A new journey	2	P32 (Woman): “Actually, there were different options in front of her, and by walking that path over and over again, she became aware of the difference between right and wrong, and by finding what was right, she put an end to her indecision.”

### 3.1 Being present and acceptance

Under the general category of being present and acceptance, it was observed that many participants expressed the ability to release emotions that felt burdensome to them and described facing the emotional weight they wished to let go of. Being present and acceptance were combined in the present analysis since participants’ reflections frequently involved being in the present moment together with openness and acceptance toward their experiences, resulting in substantial conceptual overlap between these processes during the labyrinth activity. Two prominent subcategories emerged within this category: emotional relief and emotional confrontation.

#### 3.1.1 Emotional relief

A significant number of participants (*n* = 25) described experiences of emotional relief during the labyrinth activity. Participants frequently reflected on feelings of hopelessness, disappointment, anxiety, and sadness as emotional burdens they had been carrying prior to the activity. Many participants reported feeling lighter after this process and used expressions such as freedom, relief, and reduced emotional weight when describing their experiences. These accounts suggest that the symbolic act of externalizing and reflecting on difficult emotions was associated with perceived experiences of emotional relief during the activity. These experiences were interpreted within the being present and acceptance dimension since participants appeared to engage with their emotional experiences throughout the labyrinth journey. Their reflections suggested that emotional relief emerged not simply from expressing emotions, but from attending to these experiences in the present moment and allowing them to unfold within the symbolic process of the labyrinth. Participants frequently described feeling lighter, freer, or less burdened after mindfully engaging with their emotions, suggesting that relief was associated with present-moment awareness and acceptance of their ongoing emotional experience. The following quotations illustrate how participants described these experiences throughout the labyrinth journey.

**Figure 2:**
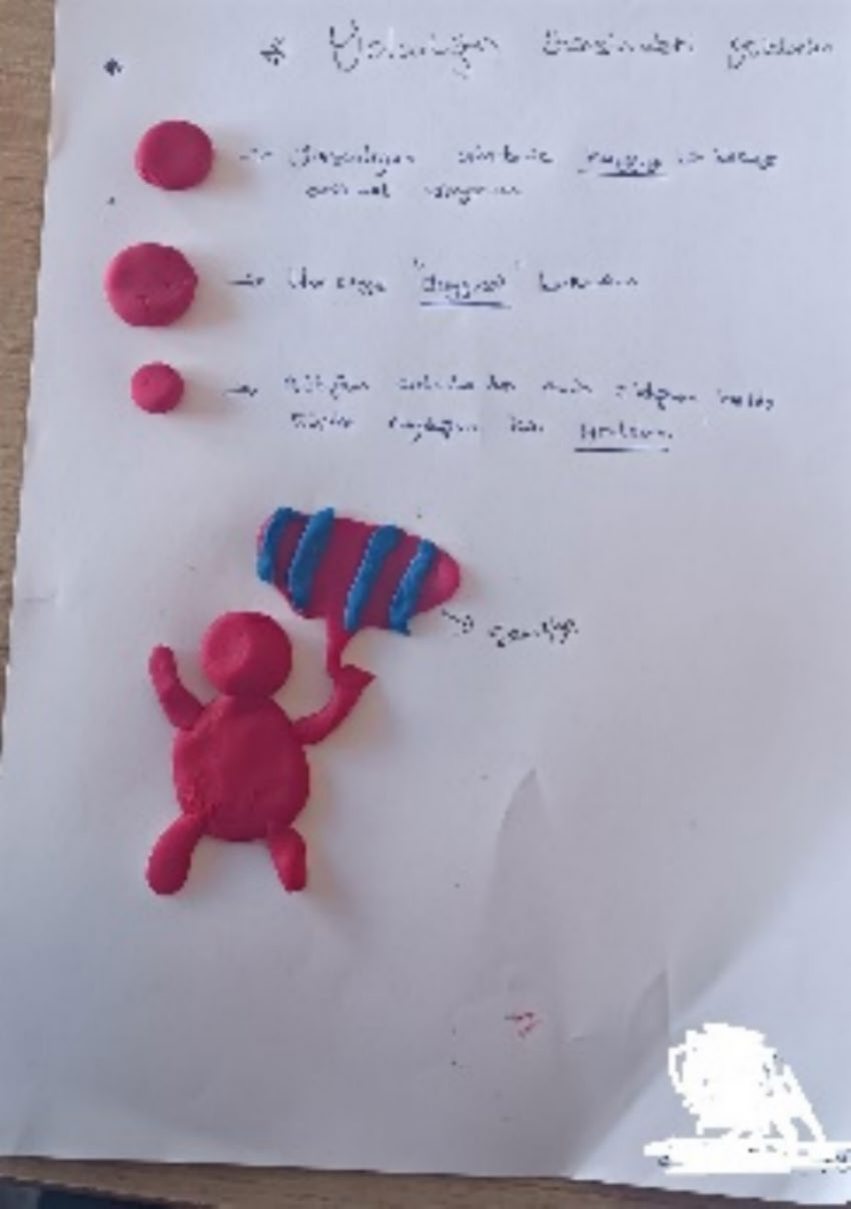
Stones, puppet, and umbrella figure of P42.

P6 (Woman), who reported feeling relieved when looking back on the burdens she had left behind during the journey, stated: *“I named my puppet ‘The Successful Rabbit.’ The stones I carried on my shoulders before starting the journey were upsetting. At the very beginning of the path, I felt deep hopelessness. One of my stones was sorrow; the others were hopelessness, intense anxiety, and failure. As I began the journey and threw one stone off my shoulder, leaving it behind on the path, I felt lighter. I felt better and better, and when I reached the final point of the labyrinth, I was so happy to look back and realize I had left those feelings behind. That really relieved me (.).* P42 (Woman), who described feeling “as light as a bird” after leaving behind her burdens during the journey, stated: “*Before the journey, I wanted to put aside the academic anxiety I had been carrying. I was tired of looking at everything emotionally… even when I was sure about my steps, I would still doubt myself. I felt lighter. You could really tell! I continued the journey with my umbrella and remaining burdens. After a while, I began to think about my other burdens too. Could I let those go as well? The first burden I left behind was the exhaustion of reacting emotionally to everything. Just the idea of letting go felt good (…). I kept walking. My load had decreased, but I wanted to be completely free. Because this feeling of lightness was something else. After a little more walking, I let go of my final burden. I wanted to step forward without being caught in doubt. Oh, finally! I got rid of the burdens. I felt light as a bird. Then I continued and rested. I congratulated myself- I rested for a long time…”* The figure displaying P42’s puppet clay, stones, and umbrella is presented below as Figure 2.

#### 3.1.2 Emotional confrontation

Within the subcategory of emotional confrontation, the majority of participants (*n* = 63) described confronting and naming emotions that they symbolically released during the labyrinth activity. Rather than avoiding unpleasant emotions, they remained present with these feelings, experienced their weight, and described placing them into the labyrinth mindfully. This process involved not denying emotions but rather engaging in emotional exposure and meaning-making through symbolic forms. These experiences were interpreted within the being present and acceptance category since participants demonstrated a willingness to acknowledge and remain in contact with difficult emotions rather than avoid or suppress them. The act of identifying, naming, and symbolically engaging with emotions such as guilt, sadness, and loneliness reflected both present-moment awareness of internal experiences and acceptance of these experiences as they occurred. The following quotations illustrate how participants acknowledged, reflected upon, and described emotions such as guilt, sadness, and loneliness during the labyrinth activity.

P3 (Woman) described and reflected upon feelings of guilt that emerged in relation to balancing her professional career, academic training, and family responsibilities: “*Between guilt and responsibility… I got on the ship too, and with me, I carry this great burden of guilt. I already feel like I neglect my 3-year-old daughter because I work. Now that I’m also studying (a degree in Psychological Counseling and Guidance), we spend even less time together, and that creates an overwhelming sense of guilt. I constantly feel sadness for coming to school when I should be with her…*” P18 (Woman) described feelings of exclusion, anger and resentment associated with her experiences: “*The unhappy brunette… Since she was little, in every environment she entered, she felt excluded and as if she were left outside the door. (.) Her biggest wish is for her mother to love her and say, ‘my beautiful girl.’ (.) Anger stone: ‘I am very angry at the people who make comments about my appearance.’ Resentment stone: ‘I’m resentful toward many family members because they mocked me for my skin color and appearance.’…”* P60 (Man) described his reflections on confronting the feeling of compassion, represented by the white stone, while also emphasizing that he did not want to leave this feeling behind: “*White stone (compassion): ‘No matter what happens, I will always be with you despite everything.’ (…). After leaving my anger behind, I was left alone with my compassion, represented by the white stone. (…). I arrived at the center together with it. I did not want to leave my compassion there. No matter how difficult it may be to be overly compassionate, I carried that feeling back on my shoulders just as I had left it at the center.*”

**Figure 3:**
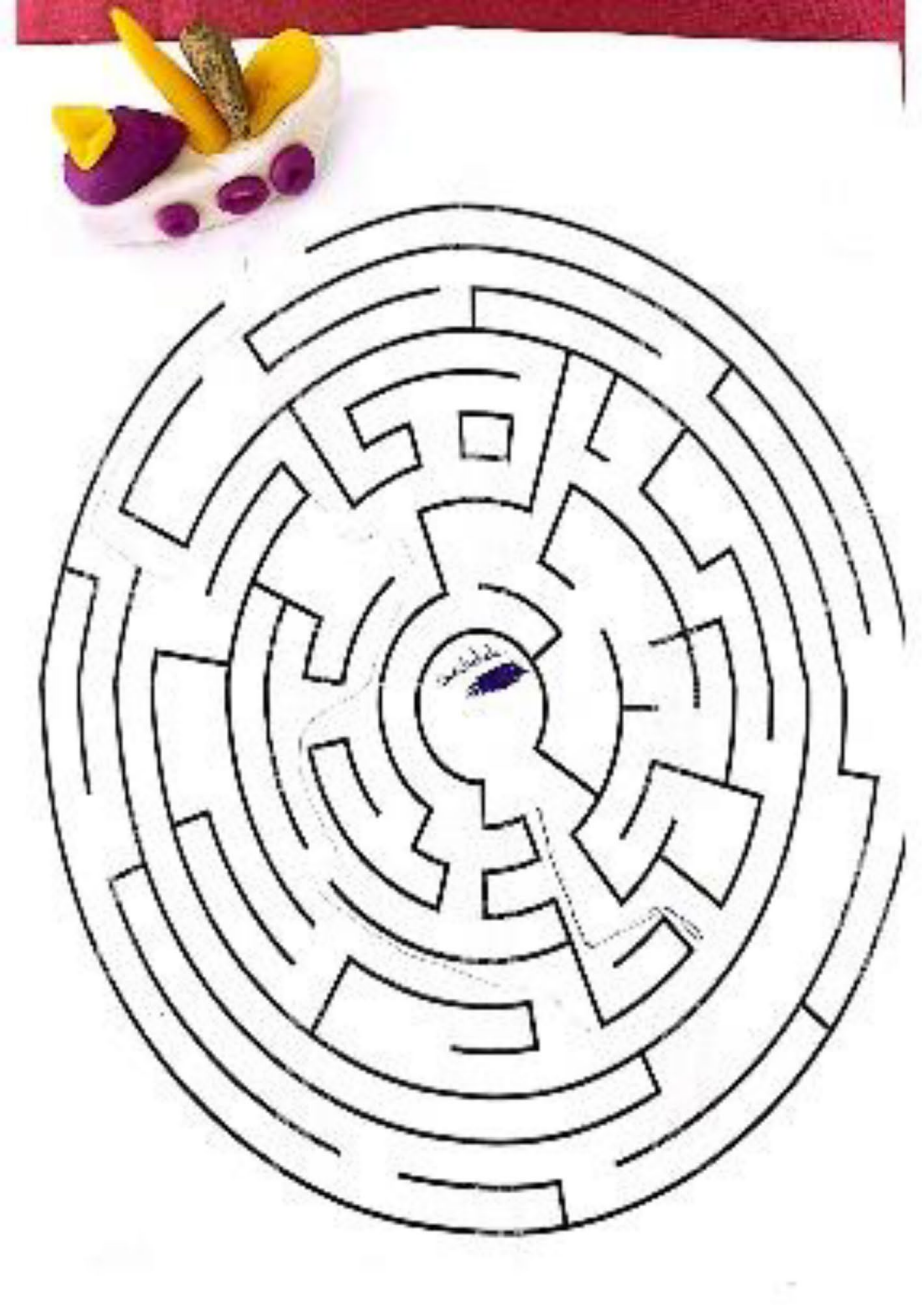
P3’s ship-shaped puppet and labyrinth.

**Figure 4:**
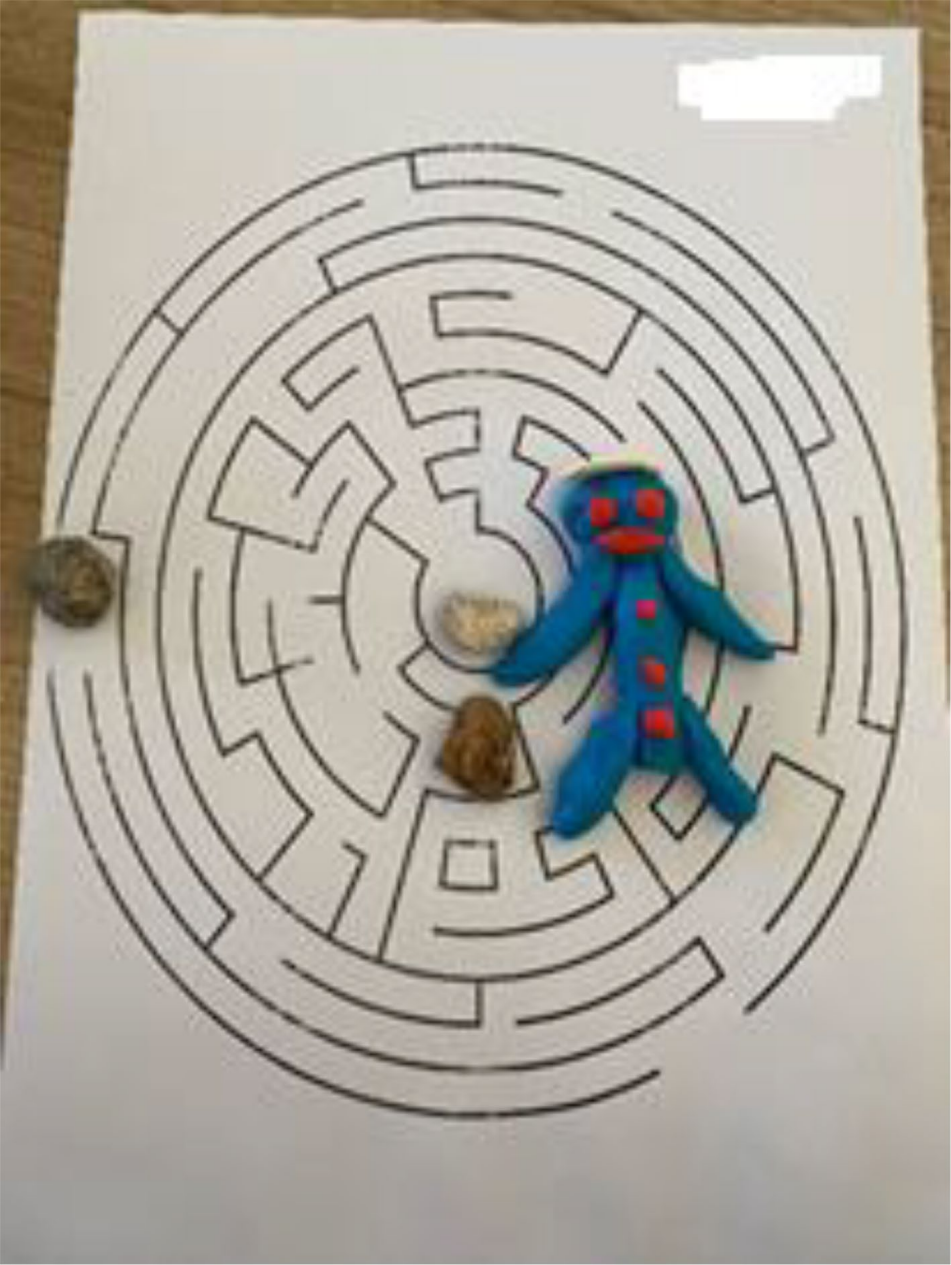
P60’s puppet and labyrinth.

The ship-shaped puppet created by participant P3 and her labyrinth are displayed below as Figure 3, and the puppet of participant P60 and his labyrinth are shown below as Figure 4.

### 3.2 Self-as-context

Within the scope of self-as-context, three subcategories were identified as self awareness, self-esteem and inner observer, and reconciliation with personal experiences and the self.

#### 3.2.1 Self-awareness

A portion of participants (*n* = 10) described experiences of self-awareness while reflecting on their emotions during the art-based labyrinth activity. While describing the emotions they experienced during the activity, some participants also reflected on previously held beliefs and perceptions about themselves. These experiences were interpreted within the self-as-context category since participants reflected not only on their emotions but also on their beliefs, self-perceptions, and personal experiences in relation to others. These reflections suggested an increased awareness of the self within a broader personal and relational context, as well as an ability to view personal experiences from a wider perspective. Such reflections contributed to the development of self-awareness during the labyrinth journey. The following quotations illustrate how participants described self-awareness while reflecting on and symbolically releasing their emotions during the labyrinth journey: P37 (Man): “*Right now, I feel stressed. Considering my age (24) and life responsibilities, I’m experiencing a bit of disappointment, a bit of sadness, and a bit of stress.* Disappointment: *‘We could have done better.’* Sadness: *‘Some of the things we imagined did not happen. We are sad, but we did not fall apart (…) I asked myself: What stage am I in? What emotions am I experiencing? (.) On my way back from the center, the first emotion I encountered was disappointment. When I saw it, I tried to transform it, but I could not do much. Sometimes, when there’s nothing you can do, you just need to leave it as it is, without trying to erase it. That can become a source of strength and motivation. So I’m leaving a bit of disappointment and bitterness inside me…*.”

**Figure 5:**
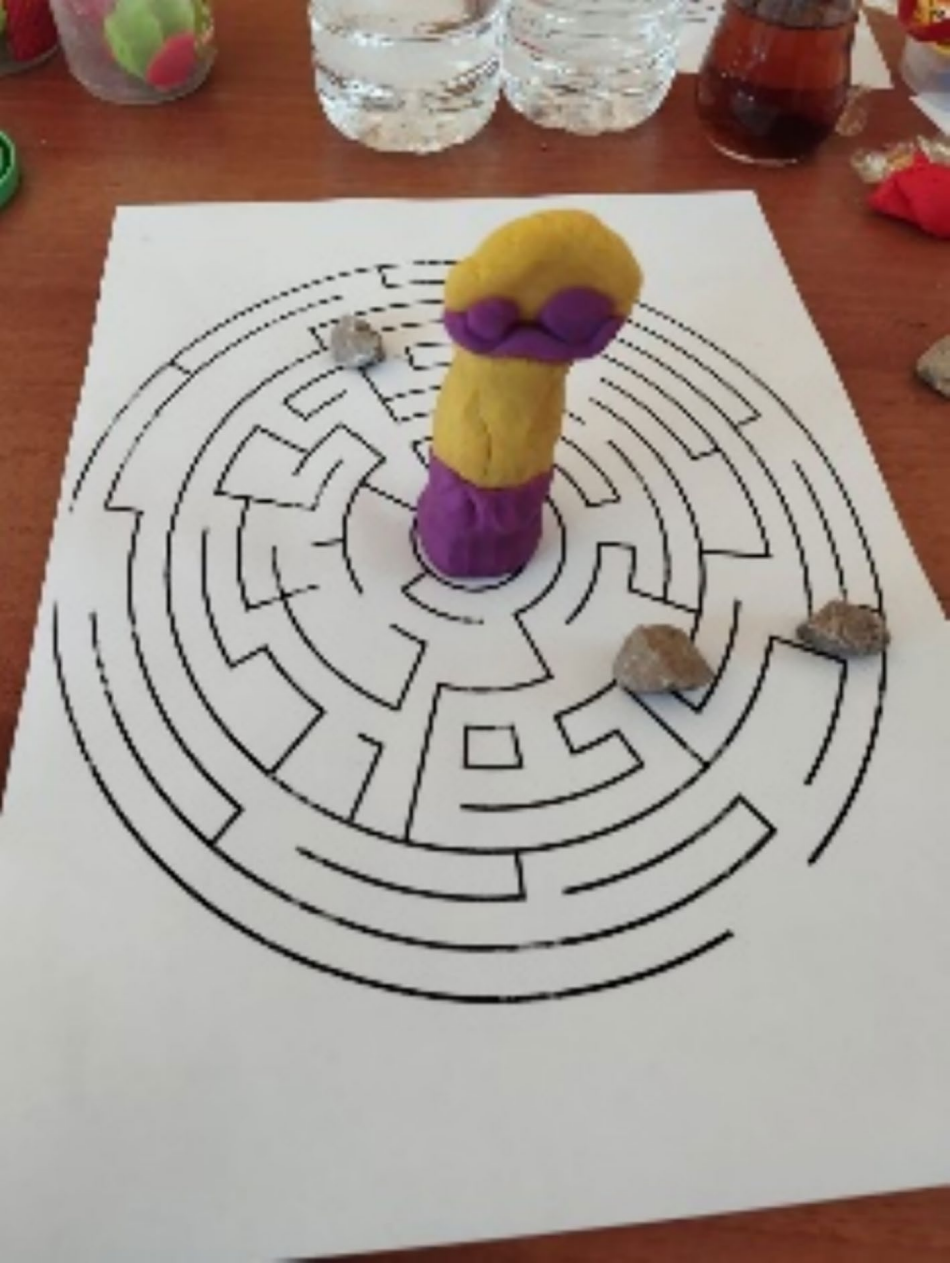
P37’s puppet and labyrinth.

P31 (Woman) reflected on becoming aware of the need to respond more calmly to situations she encounters and stated: “*Irritability (getting angry quickly at someone I love). There’s no need for that much anger. During this journey, I felt that I need to be calmer when facing situations. The reason I titled this journey ‘Pull Yourself Together’ is because I’m not that version of myself who constantly wears herself down. I’m not the version of myself who keeps draining and exhausting herself…”* P35 (Man) reflected on recognizing emotions that he had experienced but had not previously acknowledged: *“This activity helped me* become aware of *emotions I had experienced but acted as if I had not. I realized that I had found myself. This was about someone I really care about…*” The puppet of participant P37 and his labyrinth are shown below as Figure 5.

#### 3.2.2 Self-esteem and the inner observer

Among the participants (*n* = 25), many reflected on themes of self-confidence and personal strength during the labyrinth journey. The participants’ accounts frequently involved recognizing personal strengths, self-confidence, and previously overlooked qualities, suggesting an ability to observe and evaluate aspects of the self. Such reflections indicated the presence of an inner observing perspective and were associated with enhanced self-esteem and self-appreciation during the labyrinth journey. P14 (Man) reflected on the challenges encountered during the labyrinth journey and described these experiences in relation to self-confidence: “*As the labyrinth narrowed, the paths became more difficult. A wrong turn could have prolonged everything, even sent me back to the very beginning. But I did not give up. As I got to know myself and my confidence grew, I started to choose my paths more carefully. (…) This labyrinth gave me the message that every difficulty, every wrong path, and every return made me a stronger and wiser person…*.”

P9 (Woman) expressed the view that engaging in self-care from the perspective of an observer of herself could contribute positively to her self-esteem: “*The name of my journey: ‘My Own Path’…(…). I realized that this was an anger directed at myself because I had postponed things I wanted to do and had sometimes been unfair to myself… On my way back from the labyrinth, I transformed all three of these emotions- fatigue, anxiety, and self-directed anger. I replaced ‘fatigue’ with ‘rest’ and ‘renewed energy.’ I am aware that with that energy, I have achieved many things. I transformed my ‘anxiety’ into ‘self-confidence,’ because I noticed that my confidence decreases when I feel anxious. Finally, I transformed the ‘anger toward myself’ into a feeling of ‘self-love,’ because only if I love myself more can I make my life more livable….”*

#### 3.2.3 Reconciliation with personal experiences and the self

A small number of participants (*n* = 5) expressed themes of self-forgiveness and reconciliation with their personal traits or difficult emotions during the labyrinth journey. These expressions reflected a process of making peace with the self and past experiences. These experiences were interpreted within the self-as-context category since participants reflected on themselves and their past experiences from a broader perspective. Their reflections suggested an ability to separate distressing emotions, personal characteristics, and past experiences from their broader sense of self, which appeared to facilitate self-forgiveness and reconciliation during the labyrinth journey.

P14 (Man) reported experiencing regret regarding a past event and stated that, through the labyrinth activity, he had taken a step toward reconciling with himself: “*This stone represented an incident where I let a friend down in the past. A few years ago, a dear friend of mine was going through a difficult time. I knew he needed me. But during those days, I was so focused on my own problems that I could not hear his cry for help. (…) Later, I learned how deeply affected he was by his loneliness, and I felt a deep sense of guilt inside me. I took the modeling clay in my hand and made a figure. This figure represented the innocent and strong part of me that I had kept hidden. (…) I realized: guilt, when given meaning, can change. The stone was no longer a barrier- it became a symbol of forgiveness and transformation. Guilt had been replaced by forgiveness; I was now able to look ahead. Eventually, I approached the center. There, I found the true meaning of my life: making peace with myself, letting go of the burdens of my past, and forming deeper connections with my loved ones. The center was a symbol of peace and self-awareness(…).”* P18 (Woman) indicated that she had decided to be more compassionate toward herself, reconcile with herself, and foster self-love rather than continue treating herself harshly. She expressed the following views: “*This activity gave me the opportunity to see just how unfair I had been to myself. While I could accept others unconditionally, with all their differences, I realized how harsh I was when it came to myself. Accepting all of these negative emotions was very painful. But as my emotions began to transform into more positive ones, I decided to love myself, show myself compassion, and make peace with all my different sides*.”

### 3.3 Values

Some participants expressed values attributed to certain people, objects, or experiences during the art-based labyrinth activity. Based on these expressions, the following subcategories emerged: valuing challenges and solutions, valuing experiences and actions related to family member, assigning value to emotions and attributing value to early childhood memories––first toy.

#### 3.3.1 Valuing challenges and solutions

A subset of participants (*n* = 27) described how, during the labyrinth exercise, they reinterpreted challenging emotions and burdens, ultimately assigning new meaning to them and emphasizing coping and growth. These experiences were interpreted within the values category since participants appeared to assign value and meaning to both challenges and coping processes during the labyrinth journey. Their reflections suggested an increased appreciation of personal struggles and the insights gained through these experiences. Participants expressed the view that both negative and positive emotions and perspectives are valuable in the process of transforming difficult emotional experiences, as reflected in the following statements:

P26 (Woman): *“After leaving behind my* sadness stone *(which I felt after the death of my father), I was finally able to leave behind my* fear stone *(fear of death, fear that something bad would happen to me). I realized that it would be healthier for me to let go of fear by becoming aware that I did not have to be sad about everything. (…) I observed myself, and most importantly, I was able to understand myself. I replaced it with excitement, because now I no longer have the fear of dying or that something terrible will happen. On the contrary, I now feel excitement, a curiosity to ask questions and move forward with enthusiasm. Instead of sadness, I saw that I can broaden my perspective. I reminded myself once again that what I went through- like my father’s death- did not happen only to me, and that others may experience it in different ways*.” P50 (Woman): “*I chose the* anxiety stone*: ‘Yes, I know, I feel bad because of you. But whether or not I carry you is up to me. Stop blaming me, and do not stop looking ahead.’ What I learned in this process is that the labyrinth actually represents our mindset. When we experience something bad, we find ourselves in a mental labyrinth that feels heavy and hard to navigate. This labyrinth is so complex that we grow weary as we walk through it. The stones and burdens on our backs make the path feel even heavier. What really matters is not the weight itself, but the meaning we assign to it*.”

#### 3.3.2 Valuing experiences and actions related to family member

A small number of participants (*n* = 3) shared emotionally stressful family experiences during the art-based labyrinth activity workshop, while also expressing an appreciation for their family members and their actions. These experiences were interpreted within the values category since participants expressed appreciation for family relationships and family members’ actions despite emotionally challenging experiences. Such reflections suggested that participants viewed these relational experiences as personally meaningful and valuable during the labyrinth journey.

P45 (Woman) described a past experience in which she reinterpreted a family member’s behavior within its cultural context and adopted an alternative perspective toward the situation. She expressed the following views: “*(.). About seven years ago, I went out with my friends in the evening after getting permission from my mother. But my older brother did not know I had gone out. (…). But he saw me outside with my friends and got really angry at me in front of them. Years later, when I think back on this moment, my initial surprise has faded. My disappointment has lessened. (…). Now, with my current perspective, I understand that my brother probably acted that way because of social pressure. He was with his own friends at the time, and now I realize they might have said things like, ‘Why is your sister out this late? Do not you have any control over her?’ I understand that my brother reacted impulsively and out of anger in that moment. I understand and forgive him, but the sadness still remains*.”

#### 3.3.3 Attributing value to early childhood memories––first toy

One participant emphasized disappointments associated with childhood experiences, including memories related to her first-grade teacher, not having toys during childhood, and broader unmet childhood needs. These experiences were interpreted within the values category since the participant appeared to preserve a valuable connection to childhood memories despite experiences of disappointment. The narrative suggested that these memories continued to hold an important place in the participant’s understanding of the self and life experiences during the labyrinth journey. In her narrative, the metaphor of the “first toy” appeared to represent the emotional significance attributed to these childhood memories and experiences: P49 (Woman): “*(…). That feeling of silence- I think it was a kind of disappointment I had in myself. A child should not have a sense of disappointment in themselves. I believe a child aged 6 or 7 should just play, eat their favorite foods, play with toys, and be with their mother and father. I never had a toy. (…). I never got to be a child—I had to become a mother to my siblings. (…). In life, even when we are small, we want to believe there is someone who will understand and believe us. (…). I feel so sad for the little version of myself that sometimes I wish I could have been there for her. The first time I ever felt lonely was this: my first-grade teacher would either beat me or make me stand in front of the trash can every day because I did not cut my nails. I guess that’s why no one talked to me. Even during recess-when only punished students had to stay inside-I sat all alone in the classroom because there was no one else around. That was the first memory I can recall about being alone. Actually, I was just a little child and did not know how to do it. (…). I’ve always told you about my firsts, because I believe firsts are always important in a person’s life. Firsts lead to feelings. On my journey, I first left behind anger, then restlessness/fear, and finally, stress*.”

#### 3.3.4 Valuing emotions

A small group of participants (*n* = 5) expressed that although the emotions they experienced during the labyrinth journey were difficult, these emotions were also valuable and served as sources of strength. A few participants also expressed value toward positive emotions felt during the exercise. These experiences were interpreted within the values category since participants described both difficult and positive emotions as valuable aspects of their personal experiences. Their reflections suggested that these emotions were perceived not only as emotional challenges but also as sources of strength and understanding during the labyrinth journey.

P8 (Woman) stated that she had developed a good relationship with her feelings of loneliness and expressed the following views: “*I embarked on this journey with my dear friends: ‘compassion,’ ‘mercy,’ ‘uncertainty,’ ‘helplessness,’ and ‘loneliness.’ Thank you for not leaving me alone on this journey. To be honest, I cannot say I got along well with all of them. But they are all quite valuable to me. (…) Helplessness is sometimes a feeling I appreciate- because it makes me stronger. That’s why I was hesitant to leave it behind. I also realized that even though I left ‘uncertainty’ at the entrance, I still experienced indecision. I really love loneliness. I want to say that, but I’m afraid my other friends (emotions) might get upset. My favorite friend is loneliness. I realized that loneliness gives me great strength too.*” P58 (Man) expressed the following view that small things can bring happiness and are valuable during the labyrinth activity: “*During the journey, the puppet encountered brand new emotions it had never experienced before. Not only did it meet them- it felt them. Especially after getting to know the feeling of ‘happiness,’ it felt more relaxed than ever. This emotion was not only pricelessly valuable, but it was also surprising how such small things could bring happiness…*.”

### 3.4 Defusion

Nearly half of the participants demonstrated experiences of either cognitive or emotional defusion during the workshop, by reevaluating the situations they encountered. Participants expressed new ways of relating to their emotions and thoughts, which were categorized into two subcategories: emotional defusion- A new perspective on emotions and cognitive defusion- A new perspective on thoughts.

#### 3.4.1 Emotional defusion––a new perspective on emotions

A significant number of participants (*n* = 33) appeared to reconstruct their emotional experiences, labeling and differentiating them through the art-based labyrinth activity workshop while staying present in the moment. Their reflections suggested an ability to identify, label, and differentiate emotions such as sadness, anger, anxiety, and resentment, while also recognizing emotional shifts toward hope, calmness, love, and happiness. Such accounts reflected a changing relationship with emotions, in which participants appeared able to observe and reframe emotional experiences rather than remain fully identified with them.

**Figure 6:**
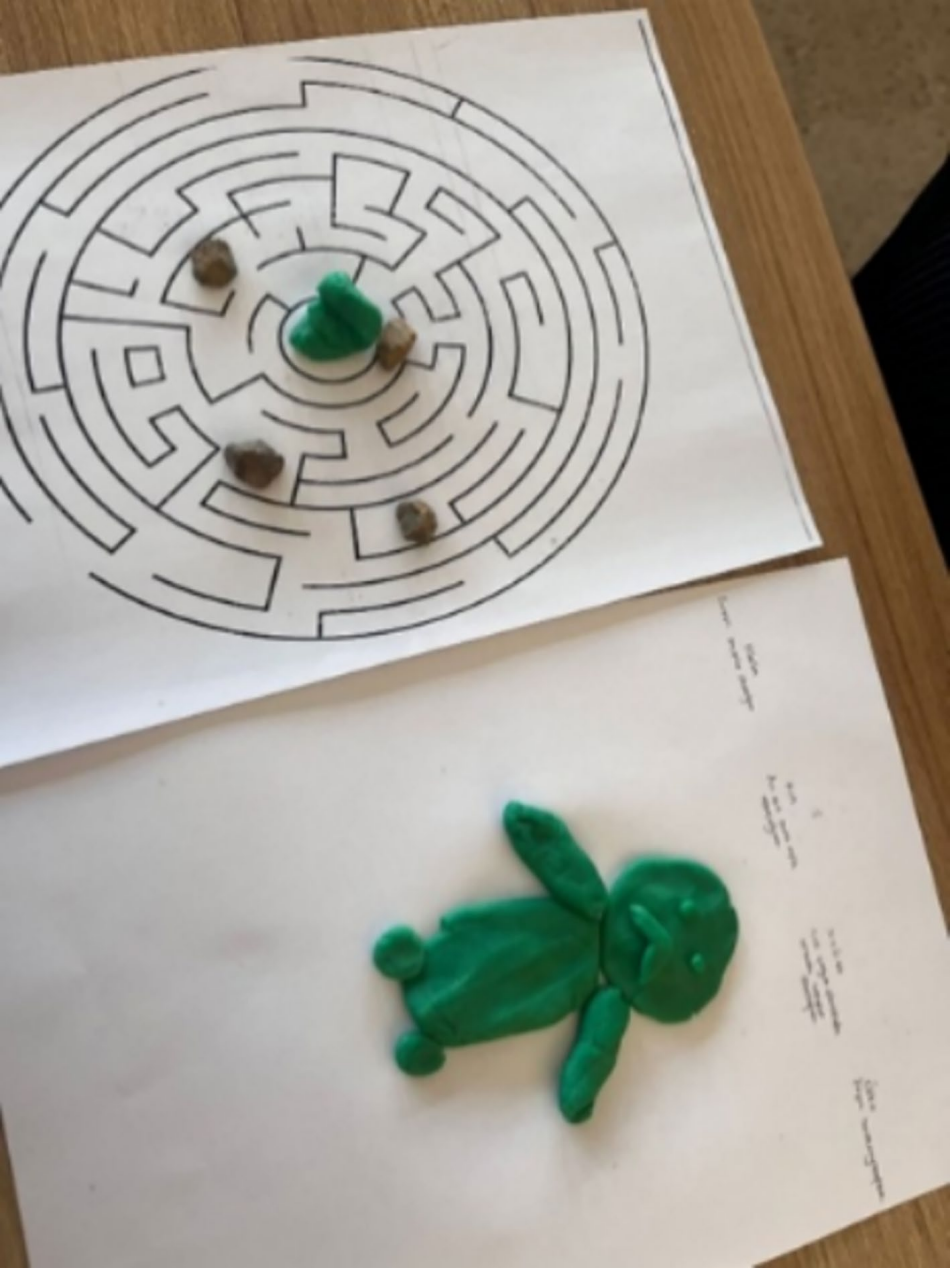
P41’s puppet and labyrinth.

P75 (Man) expressed the following views regarding the transformation of feelings of anger and sadness: “*For example, the emotion of anger transformed into love, and hatred turned into compassion. I also let go of sadness and realized that it turned into happiness. During this process, I strongly wanted to free myself from the feeling of distrust I had toward those around me. In the end, I was able to let that go too, and it turned into trust. (…) Love: ‘I will never leave you.’ Sadness and happiness: ‘Everything I have been through gives me strength.’ Compassion: ‘I am the most beautiful feeling you have ever experienced.’ Expectation: ‘Always keep me high.’*” P41 (Woman) expressed the following views concerning the transformation of anxiety into hope: *“I named this journey “the transformation of anxiety into hope.” I began this path carrying the stone of anxiety as a burden on my back. Yet, by the end of the process, that stone of anxiety had transformed into hope (…). Throughout this journey, I kept walking with the weight of that anxiety on my back, and when I reached the center, I realized that the stone was not as heavy a burden as I had thought. I could have left it there and moved on, but despite the pain it had caused me, I felt the need to transform it.(…).”* The puppet of participant P41 and her labyrinth are shown below as Figure 6.

**Figure 7:**
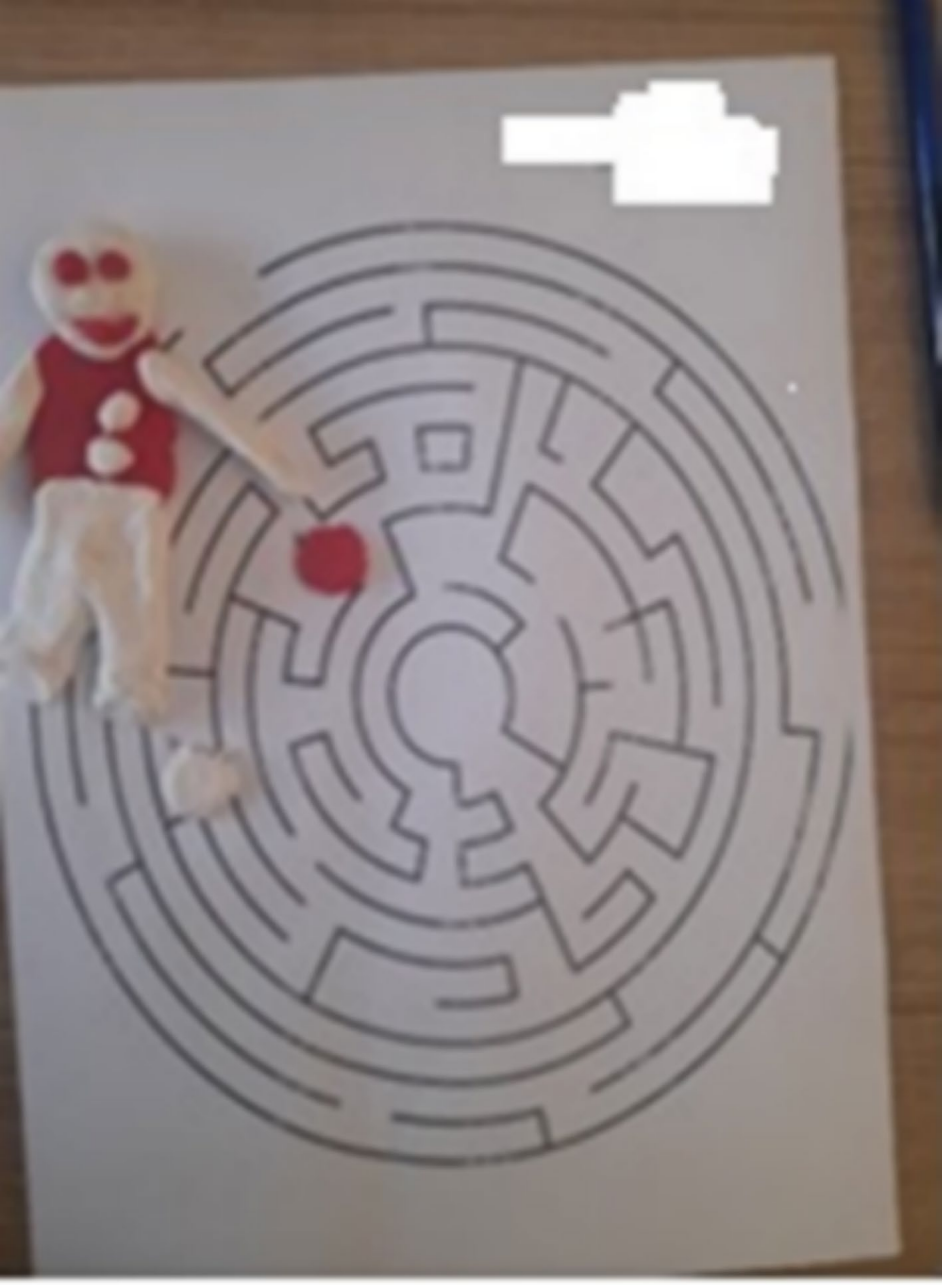
Puppet and the labyrinth of P47.

P47 (Woman), who described emotions associated with a difficult period of illness in the past, expressed the following views: *“I was diagnosed with cancer four years ago. (…). During this process, because my beliefs about dying were strong, pessimism was at the forefront. I hurt the feelings of my loved ones. My recurring thoughts and worries about whether it would come back are still with me. But as I think positively and look ahead, I believe good things will happen, and I leave the anxiety somewhere near the center of the maze. My cramps have slightly eased. That old certainty is no longer there. Finally, I reached the end of the maze (…). I reached the center of the maze, and when I turned back, I saw that the emotions of pessimism and anxiety that caused those heavy cramps in my stomach had transformed into ‘hope.’ I’m happy that I’ve gotten rid of most of the cramps…’”* The puppet and labyrinth of participant P47 are displayed below as Figure 7.

#### 3.4.2 Cognitive defusion––a new perspective on thoughts

Some participants (*n* = 13) expressed that alongside their emotional reflections, they also reevaluated their thoughts. Participants appeared to shift toward more adaptive and emotionally congruent ways of thinking while reflecting on emotionally distressing experiences during the labyrinth journey. These experiences were interpreted within the defusion category since participants appeared able to observe and reconsider stressful thoughts from a greater psychological distance through the symbolic use of the puppet and labyrinth. The following quotations illustrate these perspectives.

P56 (Man), who reflected on concerns related to his child’s health condition and described new meanings, expressed the following views: “*I was thinking about a medical situation- my child had to undergo a minor surgery. When I first learned that the surgery was necessary, I was extremely angry. Along with this anger came intense anxiety. I kept thinking, ‘Will the surgery go well?(…). After the successful surgery and early recovery, those anxious thoughts were replaced with more positive ones. The puppet I created reflected this emotional transformation- its head was modeled smaller than before, symbolizing the reduced burden. The stone representing anxiety had now transformed into a symbol of more rational thoughts and realistic assessments. Since the unnecessary, anxiety-driven thoughts had subsided, the puppet’s head was intentionally made smaller on the return journey*.” P65 (Woman), who stated that she had shifted her sense of prejudice toward analytical thinking, expressed the following views: “Into the Depths*… There are many emotions that accompany me in life. In this journey, I chose four emotions that tend to dominate in certain situations:* anger, impatience, prejudice, and resentment. *(…) Later, somewhere in the middle of the labyrinth, I left* prejudice *behind. Yes, it had often served as a kind of shield for me—but I knew it could transform into something better. (…) Eventually, I encountered my prejudice again, and it had been replaced by* analytical thinking*. I was pleased with that change, because analytical thinking could also protect me- but in a more constructive way…*.”

### 3.5 Committed action

A small number of participants expressed behaviorally oriented reflections related to people or situations they found meaningful during the art-based labyrinth activity workshop. These expressions were subcategorized under “decisive steps” and “a new journey.”

#### 3.5.1 Decisive steps

A small group of participants (*n* = 11) described taking intentional de steps related to their responsibilities or values during the labyrinth journey. These experiences were interpreted within the committed action category since participants expressed intentions to take purposeful steps related to their responsibilities and personal values during the labyrinth journey. Such reflections suggested a movement toward value-consistent actions and decisions. P3 (Woman) expressed the following views reflecting committed action toward her future goals and plans involving her daughter: “*(…)After thinking about it, I realized that the possibility of providing her (3-year-old daughter) a better life in the future—or spending more quality time in the evenings- brings me some relief. I came to understand that I need to transform this feeling of guilt into a sense of responsibility. I can say that I left behind at least part of my burden there…*.”

P17 (Man) expressed the following views reflecting committed action in managing his work, educational, and family responsibilities: “*(…). Yellow stone: When I reached the yellow stone midway through the labyrinth, I was reminded of how exhausted I often feel due to both my university courses and my teaching duties. I realized I could reduce this burden by planning my time more effectively. I decided I need to give myself more rest. Blue stone: When I came across the blue stone, it reminded me of peaceful times spent with my family. At that point, I realized the importance of managing my time and setting priorities…*.”

#### 3.5.2 A new journey

Two participants in the study described embarking on a new symbolic journey during the labyrinth activity as they sought alternative directions and emotional growth. These experiences were interpreted within the committed action category since participants described moving toward new directions and alternative ways of responding to their experiences during the labyrinth journey. Their reflections suggested an openness to change and the development of new perspectives toward their experiences.

P26 (woman), who brought her puppet to the center of the labyrinth, reported metaphorically searching for a new path on her way back from the center, with the intention of not repeating the same emotional experiences and instead embracing new ones: “*Before beginning this journey, I carried the weight of sadness and fear on my shoulders- fear of death, fear that something might happen to me. These burdens stemmed from personal losses I had experienced, including the death of my father. (…). After leaving the center of the labyrinth, I tried to find a new path to the exit- I did not want to return by the same route I had entered with all my burdens. I set out searching for a new way, wearing my old shoes but walking forward with a new perspective. On this new path, I moved forward by learning from the past. I carried my fear with me—but with courage this time, and by learning from what had come before*.”

## 4 Discussion

Within the category of contact with the present moment and acceptance, participants frequently reflected on symbolizing and externalizing their emotions while simultaneously describing the thoughts and behaviors associated with these emotional experiences. Participants described their experiences of confronting emotions, such as anger, guilt, loneliness, sadness, and anxiety, throughout the labyrinth journey. Previous literature similarly suggests that art-based and symbolic forms of expression may provide a reflective context in which individuals engage with emotionally challenging experiences and contradictions between thoughts, emotions, and behaviors (Guangli, 2020; Haeyen et al., 2022). Consistent with ACT principles, participants frequently described remaining in contact with emotional burdens and reflecting on these experiences in the present moment during the activity (Coutinho et al., 2021; Tong et al., 2026; Zarling et al., 2015).

Participants described experiencing emotional relief and contact with the present moment during the labyrinth activity. Many reflected on feeling lighter, freer, or less emotionally burdened after engaging in symbolic emotional expression. The existing literature similarly discusses how art-based expression and mindfulness-oriented engagement with artistic processes may be associated with emotional release, grounding in present experiences, and reflective emotional processing (Boldt and Paul, 2010; Simons et al., 2025; Van Lith et al., 2021).

Subcategories related to self-awareness, self-esteem, reconciliation with personal experiences, and the inner observer were also reflected in the participants’ narratives. Some participants described recognizing their personal strengths, reconsidering previously held beliefs about themselves, and developing broader perspectives on their personal experiences. Moreover, the findings related to self-forgiveness and reconciliation with personal experiences appear consistent with previous art-based literature, in which participants described experiences associated with freedom and peace in relation to personal fears, reconnection with the self, inner peace, calmness, and internal reconciliation across diverse populations, including high school students, healthcare professionals, and refugees (Arantzamendi et al., 2021; Ely et al., 2017; No, 2024). Similar reflections have been discussed in art therapy literature concerning self-awareness, confidence, identity exploration, and symbolic self-reflection among university students and other populations (Anzules et al., 2007; Boldt and Paul, 2010; Chilton, 2013; De la Croix et al., 2011; Griffin et al., 2021; Snir et al., 2024; Sonnone and Rochford, 2020). Consequently, participants’ accounts frequently involved reflective observations about the self, personal characteristics, and emotionally significant experiences emerging through creative activity, which were interpreted as conceptually consistent with self-as-context ACT processes (Browning et al., 2023).

Participants also attributed value and meaning to the challenges, coping experiences, family relationships, emotions, and childhood memories throughout their labyrinth journeys. Several participants described emotionally stressful experiences while simultaneously emphasizing appreciation, personal significance, emotional strength, and insights related to these experiences. Previous studies have similarly discussed how symbolic and artistic reflection may be associated with meaning-making, values associated with interpersonal and contextual experiences, and appreciation of family relationships and related experiences (Bosgraaf et al., 2020; Park et al., 2023). Notably, the finding related to valuing the “first toy” memory is consistent with previous literature suggesting that symbolic art-based expression may provide a safe space for exploring and attributing personal meaning to distant childhood memories through symbolic representation (Haeyen and Hinz, 2020). The valuing of emotions observed in participants’ reflections is consistent with the art-based literature highlighting flexible thinking and symbolic reframing (del Río Diéguez et al., 2024) and appears conceptually aligned with values-related ACT processes.

The findings further suggest experiences related to emotional and cognitive defusion. Participants frequently described re-evaluating emotional experiences, recognizing emotional shifts (Arriaga et al., 2024), and approaching stressful thoughts from alternative perspectives throughout the labyrinth activity. Similar discussions in the literature suggest that symbolic and externalized forms of reflection may be associated with the reinterpretation of experiences, thoughts, and emotions, as well as perspective-taking processes (Roberts, 2019). Taken together, ACT-based art activities may facilitate the development of psychological distance from distressing thoughts and emotions (Chapman and Evans, 2020). In this regard, participants’ reflections appeared conceptually consistent with emotional and cognitive defusion processes, while also involving descriptions of emotional shifts toward hope, calmness, love, and happiness, which may be considered emotional experiences associated with psychological well-being (Czamanski-Cohen and Weihs, 2023; Ke, 2024; Liu, 2022; Richesin et al., 2021; Steinbrenner et al., 2002).

Themes.

associated with committed action also emerged in the participants’ narratives. Some participants described their intentions toward new directions and purposeful actions related to personal responsibilities and values. Previous literature has similarly discussed how symbolic and experiential practices may be associated with the exploration of personal goals, emotional shifts, and new behavioral perspectives (Smeijsters et al., 2011; Sonnone and Rochford, 2020). Committed action-related ACT processes are concerned with decision-making and action-taking in relation to personally valued experiences, goals, and life directions (Anusuya and Gayatridevi, 2025).

As the participants were prospective mental health professionals, their narratives frequently involved emotional reflection, meaning-making, symbolic expression and perspective-taking experiences. The existing literature similarly emphasizes that emotional awareness, reflective engagement, and meaning-oriented processes may be particularly relevant for individuals in the helping professions (Gupta, 2023; Hen and Goroshit, 2011; Yazıcı and Özdemir, 2023). However, the present findings should be interpreted within the context of participants’ subjective written reflections regarding a single art-based labyrinth workshop and do not constitute evidence of therapeutic effectiveness or causal psychological outcomes.

### 4.1 Limitations

In this study, an art-based labyrinth activity workshop was conducted with university students who were candidates for psychological counseling. The participants were asked to express their experiences in writing. One limitation of this study is that alternative data collection methods, such as audio recordings or observational techniques, were not employed to explore how participants made sense of their inner journeys. Considering the relatively large number of voluntary participants, written narratives were deemed the most practical data collection method. Drawing on existing research, art-based practices may facilitate the externalization of participants’ self-reflective experiences and emotions through artistic production and written expression (Shukla et al., 2022). In qualitative research, reflective writing can be used as a data collection tool, enabling participants to express and make sense of their thoughts, emotions, and experiences through self-reflection (Neupane et al., 2026).

Another limitation is the large size of the participant groups during the workshop sessions, which may have posed challenges in maintaining individual focus. To address this, the facilitator (who was also the researcher) ensured the implementation of clear and consistent instructions, guiding the participants to deeply reflect and write about the meanings they attributed to their emotional journeys. Efforts were made to maintain a quiet environment throughout the sessions. Finally, the dual role of the researcher as both the facilitator of the art-based activity and the data collector presents another limitation. To mitigate this issue and strengthen the trustworthiness of the analysis, the researcher collaborated with an external ACT expert to ensure the accuracy and consistency of the themes and categories.

The study relied solely on immediate written reflections collected following a single-session workshop activity, and no follow-up procedures were conducted to explore whether participants’ reflections and meaning-making experiences persisted over time. Moreover, the reflective prompts and symbolic structure of the activity may have shaped participants’ narratives toward themes related to emotional expression, letting go, and meaning-making experiences. Furthermore, the deductive organization of the analysis around the ACT framework may have limited attention to themes and experiences outside the ACT model. Additionally, the participants were psychological counselor candidates rather than individuals formally trained in art-based practices, which may have limited the transferability of the findings to populations with different professional and educational backgrounds. Although the workshop incorporated art-based and symbolic activities, the facilitator was not functioning as a clinical art therapist, and the workshop should not be interpreted as a structured clinical art-therapy intervention. Moreover, since the written reflections were originally produced in Turkish and later translated into English by the researcher during manuscript preparation, subtle linguistic nuances or culturally embedded meanings may not have been fully preserved in translation.

### 4.2 Recommendations

#### 4.2.1 Recommendations for future research

Based on these findings, future research should further explore how desires, future goals, personal meanings, and values related to well-being are reflected and described in different art-based workshop experiences. Mixed-method designs could also contribute to a broader understanding of participants’ emotional, cognitive, and experiential reflections related to the art-making process.

Additionally, studies may explore how emotional reflections, meaning-making processes, and value-related experiences emerging during art-based workshops are described across different groups in mental health training contexts. Future research should also descriptively examine how ACT-related processes are reflected in expressive practices using both narrative and standardized forms of data collection among psychological counselor candidates and other mental health trainees, such as social workers.

#### 4.2.2 Recommendations for practitioners

The findings suggest that art-based activities may provide reflective contexts for exploring self-confidence, personal values, and experiences related to cognitive and emotional defusion among psychological counselors. In this respect, expressive and art-based activities may be incorporated into counselor education, supervision, and training processes involving mental health professionals, such as psychological counselors and social workers, to encourage emotional expression, personal reflection and experiential exploration.

Additionally, the integration of expressive art-based practices with ACT-oriented approaches focusing on thoughts, emotions, personal values, and well-being-related reflections should be further explored (Christodoulou et al., 2021; Kim and Choi, 2025). Indeed, mental health professionals’ personal experiences and professional reflections may be expressed through expressive and art-based activities, and the inclusion of such practices in mental health training contexts has been discussed and recommended (del Río Diéguez et al., 2024; Shukla et al., 2022).

## 5 Conclusion

In conclusion, this study suggests that psychological counselor candidates described emotional, cognitive, and behavioral reflections related to their participation in an art-based labyrinth activity workshop. Participants’ written reflections included experiences that were interpreted through an ACT lens, particularly in relation to emotional awareness, acceptance, values, and self-reflection. The findings provide a descriptive account of how participants made sense of their experiences within the context of the activity. In this respect, art-based activities may offer opportunities for reflection on values, personal experiences, and meaning-making processes in counselor education. Future research may further explore these experiences across different participant groups and educational contexts.

## Data Availability

The datasets presented in this article are not readily available because the datasets generated and analyzed during the current study are not publicly available due to the sensitive nature of the qualitative data and to protect participant confidentiality and are not available upon request. Requests to access the datasets should be directed togamzemukba@yyu.edu.tr.
