# Supplementary material for: An art-based labyrinth activity workshop experience: a qualitative study on psychological counselor candidates from the perspective of acceptance and commitment therapy
Source: Front Psychol. 2026 Jul 6;17:1872185. doi: 10.3389/fpsyg.2026.1872185 (PMC13381203; doi:10.3389/fpsyg.2026.1872185)
Supplement: Supplementary file 1 [file Table_1.DOCX]

**Instructions for the Written Reflection Form**

Please respond to the following questions in writing:

- What did you experience during this activity?
- Which emotional burdens did you release, and what new feelings did you gain?
- Please describe this journey as it relates to your inner world, and give your journey a title.
- Tell this journey as a story in which you are the main character. The title you provide can also reflect your emotions and the points of change or transformation you noticed during the journey.
- Please write about what this journey means to you.

You have 50-60 minutes to complete your responses. Please write your answers individually in a descriptive and narrative style.
